# Can cardiac pressure-volume catheters improve urodynamic assessment? an ex-vivo proof-of-concept

**DOI:** 10.3389/fruro.2023.1258649

**Published:** 2023-10-13

**Authors:** Silje Ekroll Jahren, Dominik Obrist, Matthias Haenggi, Fiona Burkhard, Francesco Clavica

**Affiliations:** ^1^ ARTORG Center for Biomedical Engineering Research, University of Bern, Bern, Switzerland; ^2^ Department of Intensive Care Medicine, Inselspital, Bern University Hospital, University of Bern, Bern, Switzerland; ^3^ Department of Urology, Inselspital, Bern University Hospital, University of Bern, Bern, Switzerland

**Keywords:** urodynamics, bladder pressure-volume relation, pressure-volume catheters, overactive bladder, non-voiding bladder activity

## Abstract

**Aims:**

To explore the feasibility of using pressure-volume (PV) catheters for cystometry. These catheters are well-established in cardiovascular research for simultaneous pressure and volume measurements in the left ventricle.

**Methods:**

Urinary bladders with urethras were collected from domestic pigs for ex-vivo testing. Using a pump, bladders were filled up to 500ml at five different filling rates (15, 20, 25, 30, 35ml/min), and the resulting pressures and volumes were measured using a PV catheter. The bladder were compressed externally in three different areas (central, apex and outlet) to assess the PV catheter’s ability to detect local changes in bladder volume.

**Results:**

Bladder pressure remained below 10cmH2O for all bladder filling rates. Volume measurements were compared with the volumes instilled by the pump (ground truth), proving high reproducibility and accuracy of the PV catheter measurements up to 400ml. Using the different sensing units of the PV catheter, local changes in bladder volumes could be identified and quantified.

**Conclusion:**

The main advantage of PV catheters, compared to existing technology used in urology (e.g. conventional urodynamic testing), is the possibility to i) simultaneously measure bladder pressure and volumes and ii) identify local changes in bladder volume (e.g. caused by non-voiding contractions). Both could be useful in the clinical setting to improve the diagnosis and treatment of the Lower Urinary Tract Dysfunction (e.g. overactive/underactive bladder).

## 1 Introduction

Overactive bladder (OAB), according to the International Continence Society (ICS), is a “symptom-based syndrome suggestive of lower urinary tract dysfunction,” characterized by “urinary urgency, usually with urinary frequency and nocturia, with or without urgency urinary incontinence” (1, 2). OAB has a strong negative impact on the quality of life of affected patients. The first diagnostic step in OAB is patient history and physical examination. Urodynamics are conducted to gain further information on bladder function, if considered necessary by the urologist. Urodynamic examination can identify changes in bladder pressure consistent with non-voiding contractions (NVCs), which is termed detrusor overactivity (DO). In about 30% of OAB patients, DO is not detected: this could be due to the limited duration of the urodynamic testing and/or the inability of conventional urodynamics (i.e. based on detrusor pressure recordings) to detect localized bladder contractions. To prove this hypothesis, Drake et al. (3) used eight electrodes placed on a silicon balloon to track bladder micromotions quantified as changes in electrical resistance in women. Their results showed that bladder micromotions were higher in amplitude and frequency in women with increased bladder sensation (urinary urgency group) compared to the control group. Despite the detection of micromotions, no changes in bladder pressure recordings during urodynamics could be found in some patients (3). In a follow-up study (4), conducted in isolated rat bladders, bladder movements were detected and tracked using video data analysis but, also in these experiments, no bladder pressure changes were detected.

NVCs have been observed during the bladder storage phase in healthy bladders from different species (3, 5–11). It has been speculated that they may play a role in accommodating the increasing bladder volume. Furthermore, these NVCs were enhanced in decerebrate rats (12), suggesting a centrally controlled inhibition of this activity in physiological conditions. Despite the high prevalence and high costs for the healthcare system, we are still far from understanding the origins, pathophysiology and underlying mechanisms of OAB and NVCs. Therefore, more attention should be focused on technologies which can improve the diagnostic tools and treatment options by providing objective information (e.g. urodynamic based information), in addition to the subjective information provided by the patients (OAB being symptom-based syndrome) (13–16).

To this end, in the present work we aim to introduce pressure-volume catheters (PV catheters), established for cardiovascular research, for simultaneous recordings of pressure and volume in ex-vivo bladders. These pressure-volume catheters are normally used to quantify cardiac work using pressure-volume loops and derive diagnostically relevant information (e.g. changes in pre-load, after load and muscle contractility) (17). More importantly, especially for bladder applications, these catheters can measure changes in the total volume and separate them into the individual contributions from the different regions of the organ. As PV catheters are originally designed to measure cardiac volumes (i.e. up to 120/140 ml) the main goal of the present study was to assess whether reliable dynamic measurements can be achieved in the bladder with physiological filling volumes of 300-500ml (18).

## 2 Methods

Two urinary bladders with urethras were collected from domestic pigs (ethics approval was not required as the specimens were sourced postmortem from a local abattoir). From the abattoir until the experiments, bladders were kept wet in saline and stored at 4°C. All experiments were conducted within 48 hours after collection. Before starting the experiments the bladders were filled with saline, at room temperature, multiple times (at least three times) to restore physiological organ distensibility.

### 2.1 Pressure-volume catheters

PV catheters allow simultaneous measurements of volume and pressure and are used exclusively in cardiovascular research. The pressure sensor of PV catheters consists of a classical membrane in piezoelectric materials which produces a voltage when the membrane is deformed by pressure. The pressure sensor is typically located close to the tip of the catheter. The distance between the catheter tip and the pressure sensor was 28mm in our PV catheter (Ventri-Cath 512, Millar, Texas, US).

**Figure 1:**
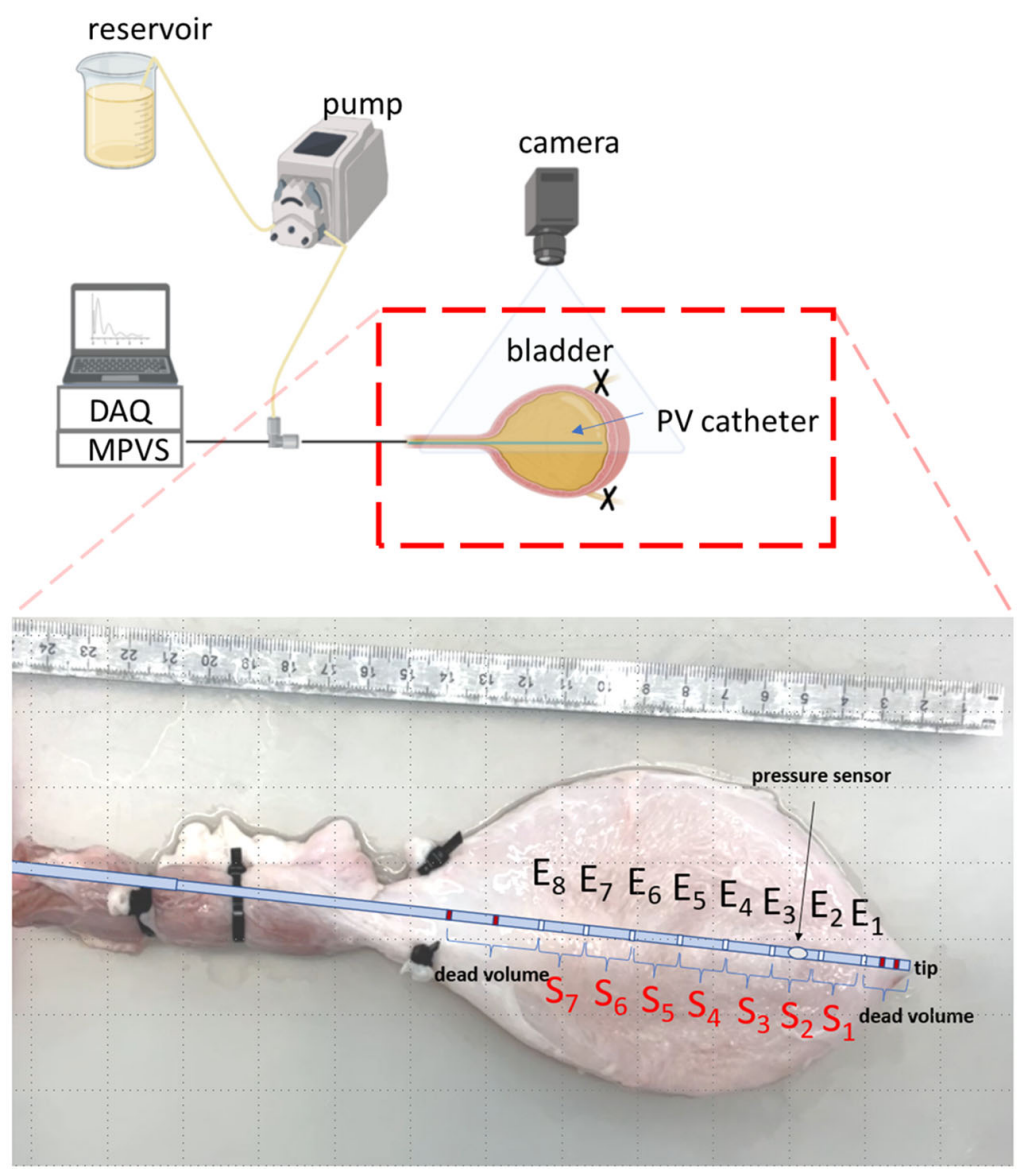
Schematic of the experimental setup (created with BioRender.com). The inset shows the positions of the different electrodes (E_i_) and sensing elements (S_i_) of the PV catheter within the ex-vivo bladder. The dark-red electrodes are the primary and secondary electrodes which are used to generate the signals with no-sensing function. Thus, the region from the tip of the catheter to the first sensing electrode E_1_ is considered ‘dead volume’.

To measure the volume with PV catheters, constant electrical currents are generated between the distal and proximal electrodes of the catheter, such that an electrical field is created. In a conductive fluid, volume changes result in changes in the electrical impedance. Between two adjacent electrodes, the electric impedance is proportional to the fluid resistivity and the distance between electrodes, while it is inversely proportional to the cross-sectional area of an ideal cylindrical volume considered at the level of the electrodes (17). Since fluid resistivity and distance between electrodes are constant in typical PV catheter measurements, the electrical impedance between two subsequent electrodes depends only on the local volume changes. The used PV catheter was characterized by 12 electrodes, four of which (dark red electrodes in **Figure 1**) were used to enforce the current (primary and secondary electrode pairs with opposing polarity generated the electrical field) with no-sensing function while all the other electrodes E_1-8_ represented the sensing part resulting in seven sensing elements (S_1-7_, **Figure 1**). The inter-space between adjacent electrodes is 12mm. The distance between the catheter tip and the first sensing electrode E_1_ was 10mm, the volume included in this region could not be measured (dead volume, **Figure 1**). Additionally, volume above sensing electrode E_8_ could not be measured either (bladder neck) and might create a second area of dead volume depending on the size of the bladder.

### 2.2 Experimental setup and protocol

#### 2.2.1 Bladder filling and emptying

A roller pump (Model No: LabF1-III, Shenchen, China) was used to fill the bladder with saline at five different flow rates (Q=15, 20, 25, 30, 35 ml/min) from 0 (empty bladder) to 500ml. To avoid retrograde leakage, both ureters were closed using cable binders. The PV catheter connected to a MPVS Ultra console (Millar, Millar, Texas, US) was inserted in the bladder and it was used to measure bladder pressure and volume signals during filling. The signals were acquired (Powerlab, ADInstruments, Dunedin, New Zealand) and recorded on a laptop using LabChart Pro (ADInstruments, Dunedin, New Zealand). The sampling rate was 1000Hz. Bladders were placed inside a tray and completely emptied before each filling. A three-way Luer connector was used to connect the tube from the roller pump with the PV catheter introducer sheath, resulting in a single tube (with the PV catheter in its lumen) inserted in the urethra: the same line could therefore be used to fill the bladder and for pressure-volume measurements (using the PV catheter) (**Figure 1**). A camera was mounted to have a top view of the bladder during bladder inflation. Videos were recorded at 30 frames per second. In this study, we define the total bladder volume (TBV) as the volume of the whole bladder which is the sum of all local bladder volumes (LBVs). These LBVs were measured by each sensing unit S_i_ (i.e. volume between two subsequent electrodes, **Figure 1**).

**Figure 2:**
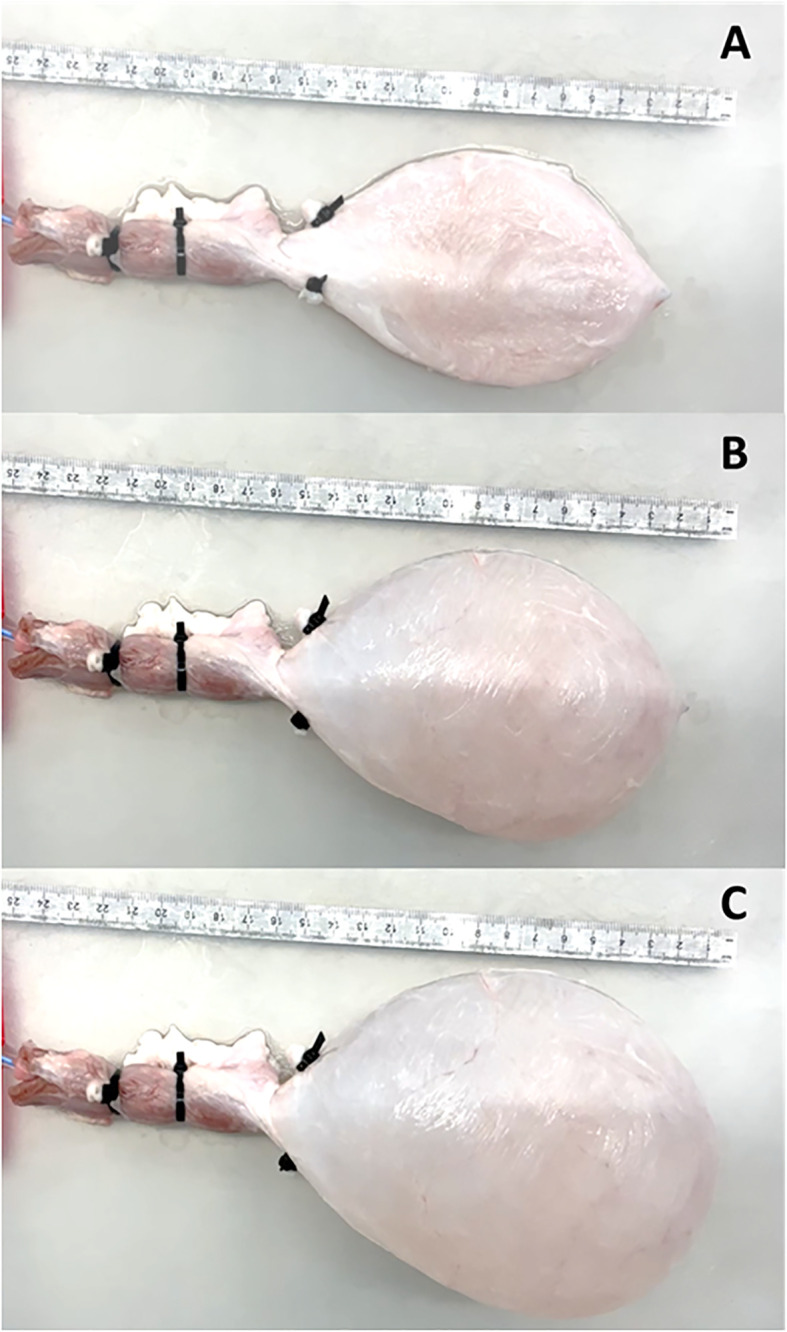
Sequence of bladder filling at: **(A)** 0ml, **(B)** 250ml and **(C)** 500ml.

In all experiments the pump was switched off when the bladder volume reached 500ml (**Figure 2**). To test reproducibility of the PV catheter recordings the bladder was filled twice at a rate of Q = 35ml/min. The bladders were emptied, using the same setup, with Q=-35ml/min by reversing the sense of rotation of the roller pump.

#### 2.2.2 Bladder local compressions

**Figure 3:**
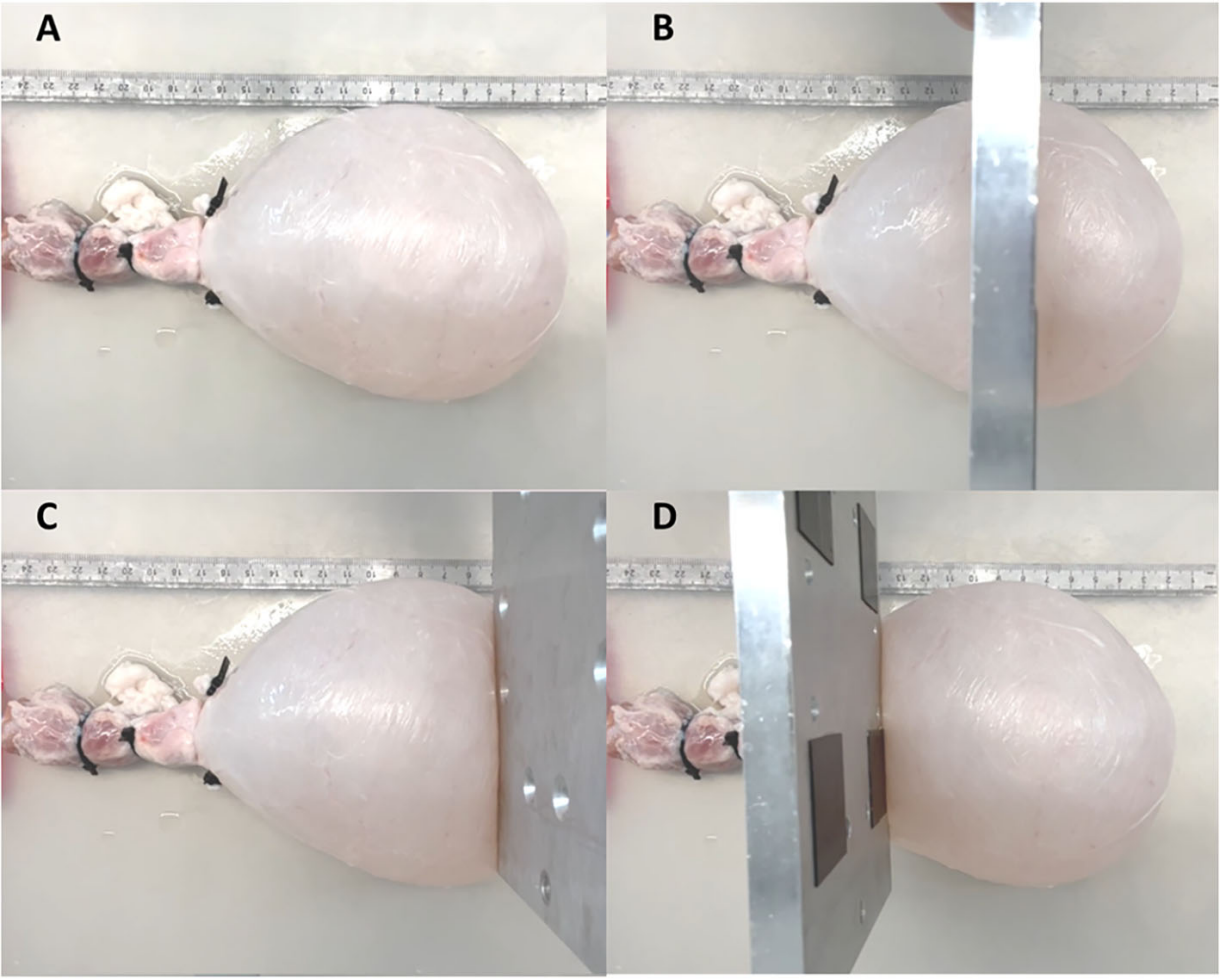
Bladder was filled until 300ml **(A)**. A metal plate was used to compress the bladder at central **(B)**, apex **(C)** and outer **(D)** region.

To test the ability of PV catheters to detect local changes in bladder volume (**Figure 3A**), we used a metal plate (10mm thickness) to compress the bladder in three different areas: central (**Figure 3B**), apex (**Figure 3C**) and outlet (**Figure 3D**). In these compression tests, the bladders were filled with 300ml of saline, and the PV catheter was used to record bladder pressure and volume (see **Supplementary Video**).

#### 2.2.3 Calibration of bladder volumes

The integral of the given flow rate (provided by the roller pump) was taken as ground truth for the bladder volume. This bladder volume was used to calibrate and convert the measured voltage signals from the PV catheter (V) into volumes (ml): a third-order polynomial was chosen to fit the measured signals of the PV catheter with the ground-truth bladder-volume to account for the non-linearity of the measured signals.

## 3 Results

### 3.1 Total bladder volume measurements

**Figure 4:**
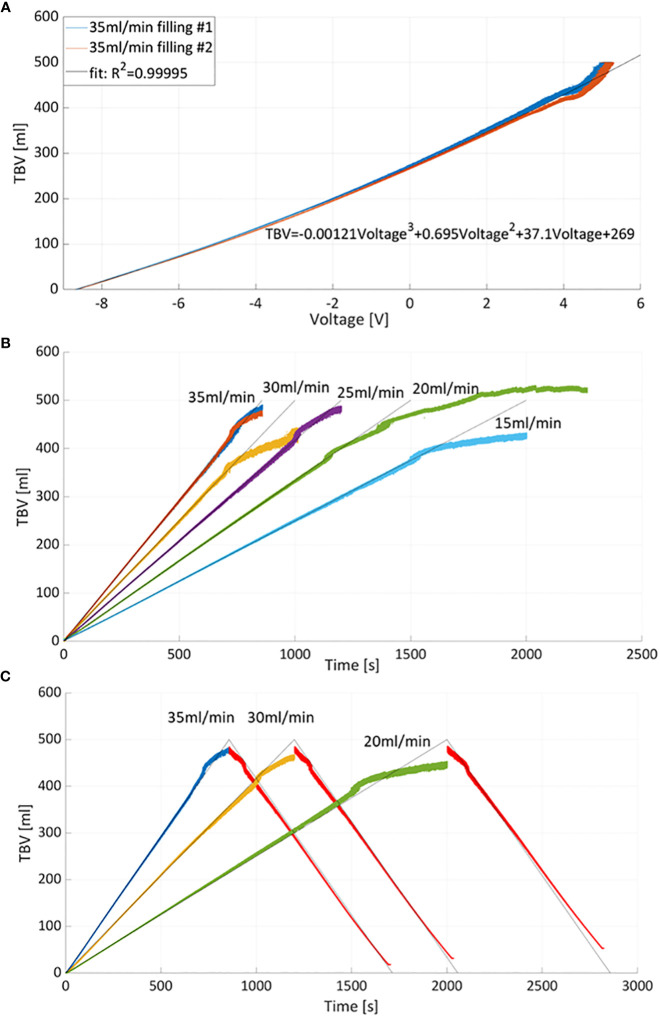
**(A)** Total bladder volume (TBV) versus measured voltage curves and interpolating curve. Two measurements of bladder filling with 35ml/min were considered for calibration. **(B)** Calibration was used to derive TBV at different filling flow rates. The grey lines correspond to theoretical volume values estimated from the flow rates while color curves correspond to TBV measured by PV catheter. **(C)** Example of bladder filling and emptying cycles: bladder emptying was obtained by reversing the pumping with constant flow rate equal to 35ml/min (red curves).

Bladders were filled twice at a constant flow rate (Q=35ml/min) and associated PV catheter voltages were measured. **Figure 4A** shows the repeatability between the measurements (blue curve versus red curve). The average voltage of the two measurements was considered for the third order polynomial regression which resulted in R-Squared (R^2^)=0.999 (higher order polynomials was discarded as the third-order polynomial resulted in R^2^~1). The regression was used to convert voltages into total bladder volumes (TBV). Calibrated TBV measurements, during bladder filling (**Figure 4B**) and emptying (**Figure 4C**), were compared with the theoretical total bladder volumes derived from the associated pump flow rates (grey lines in **Figures 4B, C**). In general, PV catheter measurements showed high levels of accuracy up to 400 ml. The maximum residual for all points was 10ml for 0<TBV<400ml. Above 400ml the PV measurements deviated from the theoretical values and the maximum residuals reached 30ml. The explanation of the possible cause is provided in the next paragraph.

### 3.2 Local bladder volume measurements and compression tests

**Figure 5:**
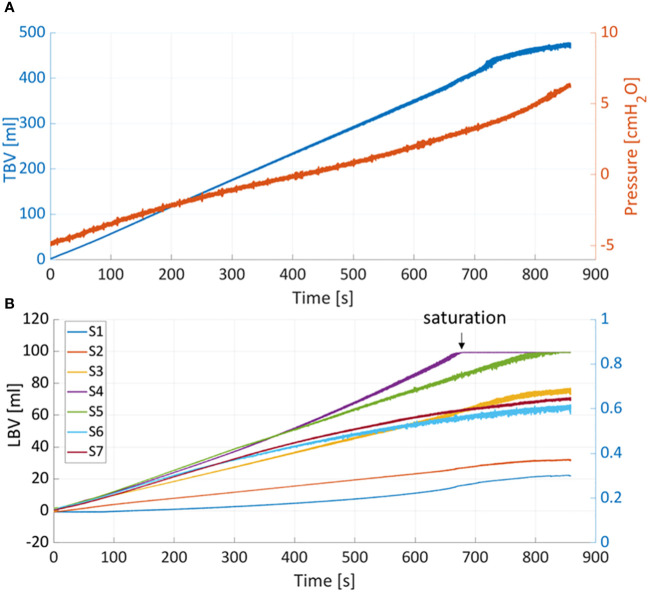
**(A)** Total bladder volume (TBV) and pressure measured with PV catheter during filling at 35ml/min. **(B)** TBV is divided into the local bladder volumes (LBV) according to corresponding sensing elements (S_1-7_).

**Figure 6:**
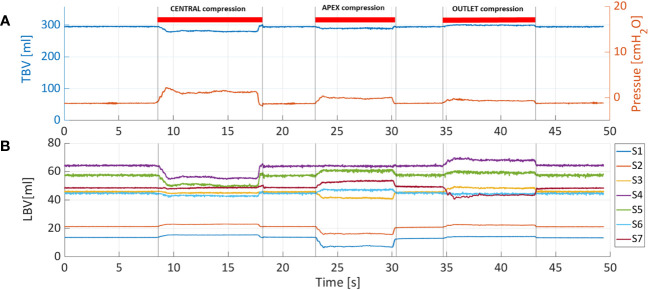
**(A)** Total bladder volume (TBV) and pressure were measured with PV catheter at fixed bladder volume of 300ml and considering three different compression sites: central, apex and outlet. **(B)** Also in this case, TBV is divided into the local bladder volumes (LBV) according to corresponding sensing elements (S_1-7_).

**Figure 5A** shows the pressure and TBV associated with a bladder filling rate of 35ml/s. The pressure remained below 7cmH_2_O throughout the filling phase (red curve). One of the main advantages of PV catheter measurements is the possibility to split the TBV measurements into the associated local bladder volumes (LBV) using the different volume/sensing elements S_1-7_ positioned from the bladder apex to the bladder neck (see **Figure 1**). **Figure 5B** shows the corresponding LBVs measurements. During the filling, the bladder expanded more in its central region than its peripheral parts, as shown by the higher values of S_4_ and S_5_ (central sensing units, **Figure 1**). At around t=680s, the S_4_ sensor values saturated (LBV of S_4_>95ml). This saturation affects the overall TBV measurements and can explain the deviation of the TBV from the theoretical values in similar cases (**Figure 4**). **Figure 6** shows the resulting changes in TBV and pressure (**Figure 6A**) and in LBVs (**Figure 6B**) associated with local compression of the bladder in the three different bladder regions (central, apex and bladder neck). Starting from a central compression, the LBV of the two central sensing elements S_4_ and S_5_ (**Figure 6B**) dropped significantly (13.2% and 12.2% decrease, respectively). Because of this compression, the liquid inside the bladder moved in two directions: towards the apex (leading to increases in S_1_ and S_2_) and towards the outlet. For the latter, since the liquid could move beyond the last sensing element (S_7_), towards bladder neck and urethra, no increase was detected in S_6_ and S_7_. This also explains the sudden drop in TBV in **Figure 6A** as some volume is ‘lost’ outside the sensing region of the PV catheter towards the bladder neck and towards the ‘dead volume’ in the bladder apex. Mass conservation within the measured domain is therefore not satisfied due to this loss in volume. A pressure increase of approximated 3.5 cmH_2_O is recorded (red curve in **Figure 6A**). During apex compression, liquid moves away from the apex, towards the central and outlet area. This explains the volume drop of S_1_, S_2_ and S_3_ and the increase in S_5_, S_6_ and S_7._ Also, in this case some volume is ‘lost’ because it moves beyond S_7_, and total mass is not conserved (small drop in TBV). During the outlet compression, the liquid mainly moves towards the central and apex regions: signal from S_7_ drops while it increases in the remaining sensing elements. In this case, the volume loss outside the sensing regions is the smallest as it mainly involves the small region without sensing elements close to the apex of the bladder (dead volume in **Figure 1**). For this reason, the TBV stays almost constant.

## 4 Discussion

The present study is, to our best knowledge, the first attempt to use PV catheters for urological research. These catheters are designed to measure ventricular volumes which are considerably smaller than typical bladder filling volumes. In addition to the obvious advantages of ensuring simultaneous measurements of pressure and volume, the main advantage of PV catheters is the possibility to split the total bladder volume (TBV) measurements into the analysis of local bladder volumes (LBVs). This could possibly enable the detection of Non-Voiding Contractions (NVCs) in overactive bladder (OAB) patients which do not lead to changes in pressure. The primary objective of the present study was therefore to investigate the feasibility and accuracy of using cardiac PV catheters for simultaneous recordings of pressure and volumes in ex-vivo porcine bladders. The secondary objective was to measure LBVs and investigate if local compressions could be clearly identified.

**Figure 7:**
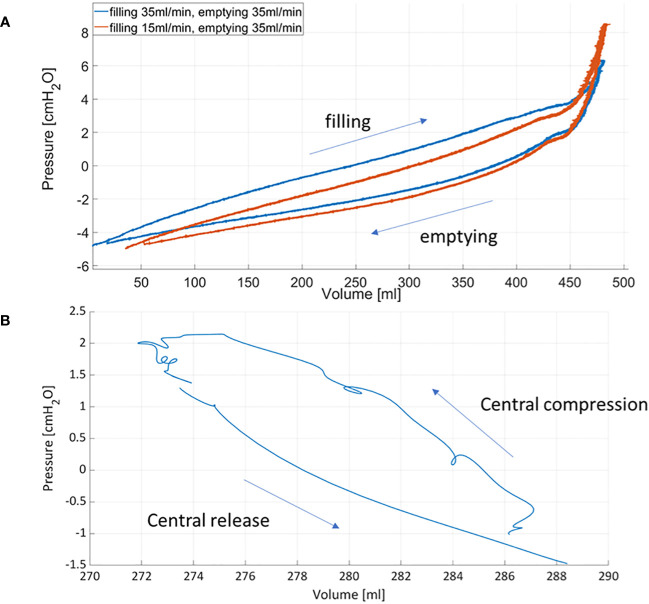
Pressure-volume curves obtained during bladder filling and emptying using the roller pump **(A)** and after compressing and releasing the central region of the bladder **(B)**.

The PV catheter was able to provide accurate measurements of bladder filling and emptying up to 400ml volumes and with simultaneous bladder pressures well below 10cmH_2_O. Normal functional bladder capacity in adults is 300-400 ml (18), however it can be higher (up to 700ml in men). Since in OAB patients, urge occurs at relatively low bladder volumes, the achievable range with existing PV catheters could be considered sufficient for these patients. However, for higher bladder volumes, device optimization from the manufacturer may be required. For the clinical setting, *ad-hoc* console for signal acquisition, recording and signal display would be required to guide the clinicians by providing an overview of bladder volume, pressures and possible locations of bladder micromotions. The accurate simultaneous measurement of pressure and volume enables the direct plotting of PV loops. **Figure 7** shows the corresponding pressure-volume curves obtained during bladder filling/emptying cycle (with the pump) and when compressing the central region of the bladder.

Recent works from Peng et al. (19) and Lau et al. (20) have pointed out that, although bladder pressure and volume are measured during cystometry, relating volume changes with pressure changes during micturition has received scarce attention. Following a thermodynamic approach used for pressure-volume analysis of ventricular function, they derived the first bladder PV loops in animal models (19) and patients (20) enhancing their potential for diagnostics. In particular, they showed that pressure-volume loops could be a useful tool to identify changes in: i) bladder contractility in rats, ii) bladder pre-load (obtained with different bladder filling flow rates in rats) and iii) bladder after-load [i.e. changes in urethral resistance in rats (19) and in patients (20)]. However, in their studies the bladder volume was derived by subtracting the voided volume from the infused volume. Since bladder volumes were not directly measured, the timing and accuracy of their volume estimations could have been affected. In the present study, we show that the cardiac PV catheters can mitigate these problems enabling direct and simultaneous pressure and volume measurements.

In this study, we showed that LBV changes can be detected and accurately measured by the PV catheter. Local compressions were evidenced as reduced volumes in the corresponding sensing units (S_i_) and as increased volume in the other segments (**Figure 6**). This could potentially have clinical implications by minimizing invasiveness and improving the accuracy of established tests, as well as having an increased sensitivity for identifying muscle activity associated with OAB and possibly its exact location. This might be helpful, for example, to improve the diagnosis of OAB patients and, consequently, restrict the treatment to a specific bladder region (e.g. Botox) avoiding treating the whole bladder.

Better understanding of the pathophysiology of Lower Urinary Tract Dysfunction (LUTDs) could lead to new therapeutic alternatives, which are desperately needed.

## 5 Conclusion

We show that cardiac PV catheters are feasible and accurate in measuring bladder pressure and volume during the storage/filling and emptying phase. Compared to conventional urodynamics, PV catheters ensure simultaneous measurements of effective bladder pressure and volume. Additionally, PV catheters could enable evaluation of contractile function and identification of the area contracting, potentially improving diagnostic accuracy and sensitivity thus leading to improved and new treatment strategies.

### 5.1 Limitations

The present study represents a proof-of-concept conducted in a small number of non-viable ex-vivo bladders. Before considering the use of PV catheters in the clinical setting, further investigations in viable organs (ex-vivo and *in-vivo*) are necessary to demonstrate that localized bladder micro-motions can be detected with PV catheters. For example, isolated whole bladder experiments could be performed, similarly to Chakrabarty et al. (4): bladder micromotions could be quantified using markers (e.g. carbon particles) via image processing and, these results, could be compared with the associated pressure and volume signals from the PV catheter. In clinical settings, the presence of a residual volume of urine, at the start of the artificial bladder filling, and/or the simultaneous production of urine from kidneys can affect the accuracy of volume measurements using PV catheters. However, these limiting factors could be overcome by measuring the residual bladder volume (e.g. using an ultrasound scanner) and/or measuring the voided volume after micturition, at the end of the urodynamic procedure. Moreover, movements of the catheter within the bladder may lead to different dead volumes and, consequently, to motion artifacts. These are well-known limitations also for ventricular volume measurements (21). For this reason, PV catheters measurements in bladder could be assisted with X-rays (or ultrasound) imaging to gain experience on the measuring signals and distinguish them from motion artifacts. This could be necessary, especially in the first clinical tests (due to the pioneering nature of these measurements) to verify the correct catheter position and its possible movements within the bladder.

## Data availability statement

The original contributions presented in the study are included in the article/**Supplementary Material**. Further inquiries can be directed to the corresponding author.

## Author contributions

SJ: Conceptualization, Data curation, Investigation, Methodology, Validation, Writing – original draft, Writing – review & editing, Software. DO: Conceptualization, Methodology, Writing – review & editing, Resources. MH: Conceptualization, Methodology, Writing – review & editing, Validation, Software, Visualization. FB: Conceptualization, Methodology, Writing – review & editing, Data curation, Formal Analysis. FC: Conceptualization, Data curation, Methodology, Writing – review & editing, Funding acquisition, Investigation, Project administration, Resources, Supervision, Validation, Writing – original draft.
